# Supplementary material for: Neuronal sFlt1 and Vegfaa determine venous sprouting and spinal cord vascularization
Source: Nat Commun. 2017 Jan 10;8:13991. doi: 10.1038/ncomms13991 (PMC5234075; doi:10.1038/ncomms13991)
Supplement: Supplementary Information — Supplementary Figures, Supplementary Tables. [file ncomms13991-s1.pdf]

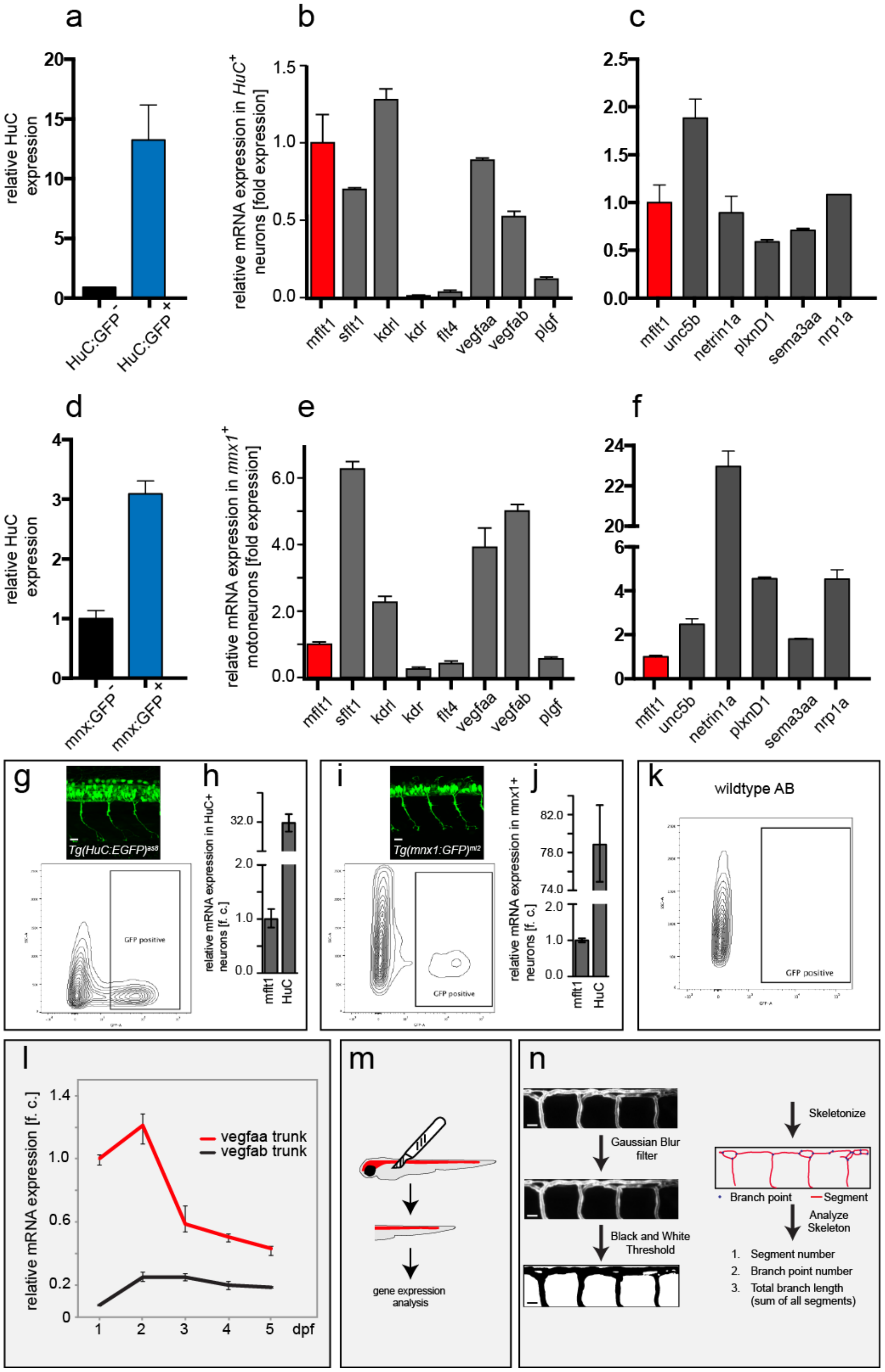

Supplementary Figure 1 | FACS of neuronal cells

(**a-f**) Neuronal cells were isolated from *Tg(HuC:EGFP)*^{as8} embryos and *Tg(mnx1:GFP)*^{ml2} embryos at 24hpf by FACS with indicated gating settings (g-l). (**a**) Expression of pan-

neuronal marker HuC in the GFP positive fraction (HuC:GFP$^+$) and GFP negative fraction (HuC:GFP$^-$) sorted from *Tg(HuC:EGFP)^{as8}* embryos. (**b**) Quantitative expression (TaqMan) of *mflt, sflt1, kdrl, kdr, flt4, vegfaa, vegfab, plgf*; (**c**) Quantitative expression (TaqMan) of *unc5b, netrin1a, plexinD1, semaphorin3aa, neuropilin-1a* in the GFP positive cell fraction FACS sorted from the pan-neuronal reporter *Tg(HuC:EGFP)^{as8}*, mean ± s.e.m, *n*=3 experiments, 60 embryos/experiment. Expression levels were normalized to the expression of *mflt1* (*mflt1* expression was set at 1.0). (**d**) Expression of pan-neuronal marker HuC in the GFP positive fraction (Mnx:GFP$^+$) and GFP negative fraction (Mnx:GFP$^-$) sorted from *Tg(mnx1:GFP)^{ml2}* embryos. (**e**) Quantitative expression (TaqMan) of *mflt, sflt1, kdrl, kdr, flt4, vegfaa, vegfab, plgf.* (**f**) Quantitative expression (TaqMan) of *unc5b, netrin1a, plexinD1, semaphorin3aa, neuropilin-1a* in the GFP positive cell fraction FAC sorted from the pan neuronal reporter *Tg(HuC:EGFP)^{as8}*, mean ± s.e.m, *n*=3 experiments, 60 embryos/experiment. Expression levels were normalized to the expression of *mflt1* (*mflt1* expression was set at 1.0). (**g-k**) Gating settings for (g,h) *Tg(HuC:EGFP)^{as8}* embryos, (i,j) *Tg(mnx1:GFP)^{ml2}* embryos. The pictures in the inset indicate the *in vivo* expression domains of (g) *HuC* and (i) *mnx1*. Note that *HuC* marks all spinal cord neurons; *mnx1* marks only the motoneuron population. (**k**) Verification of gating settings in control embryos. (**l,m**) Tails were dissected from zebrafish embryos between 24hpf and 5dpf, RNA was isolated and used for *vegfaa* and *vegfab* real-time qPCR. (l) Quantitative expression of *vegfaa* and *vegfab* in the trunk of zebrafish embryos at indicated time points; mean ± s.e.m, *n*=3 experiments, n=30 embryos/experiment. (**m**) Semi-automated vascular network analysis was performed using ImageJ with indicated plugins. Vessel segment number, branch point number and total branch length was determined. Scale bar, 25µm in g,i,m.

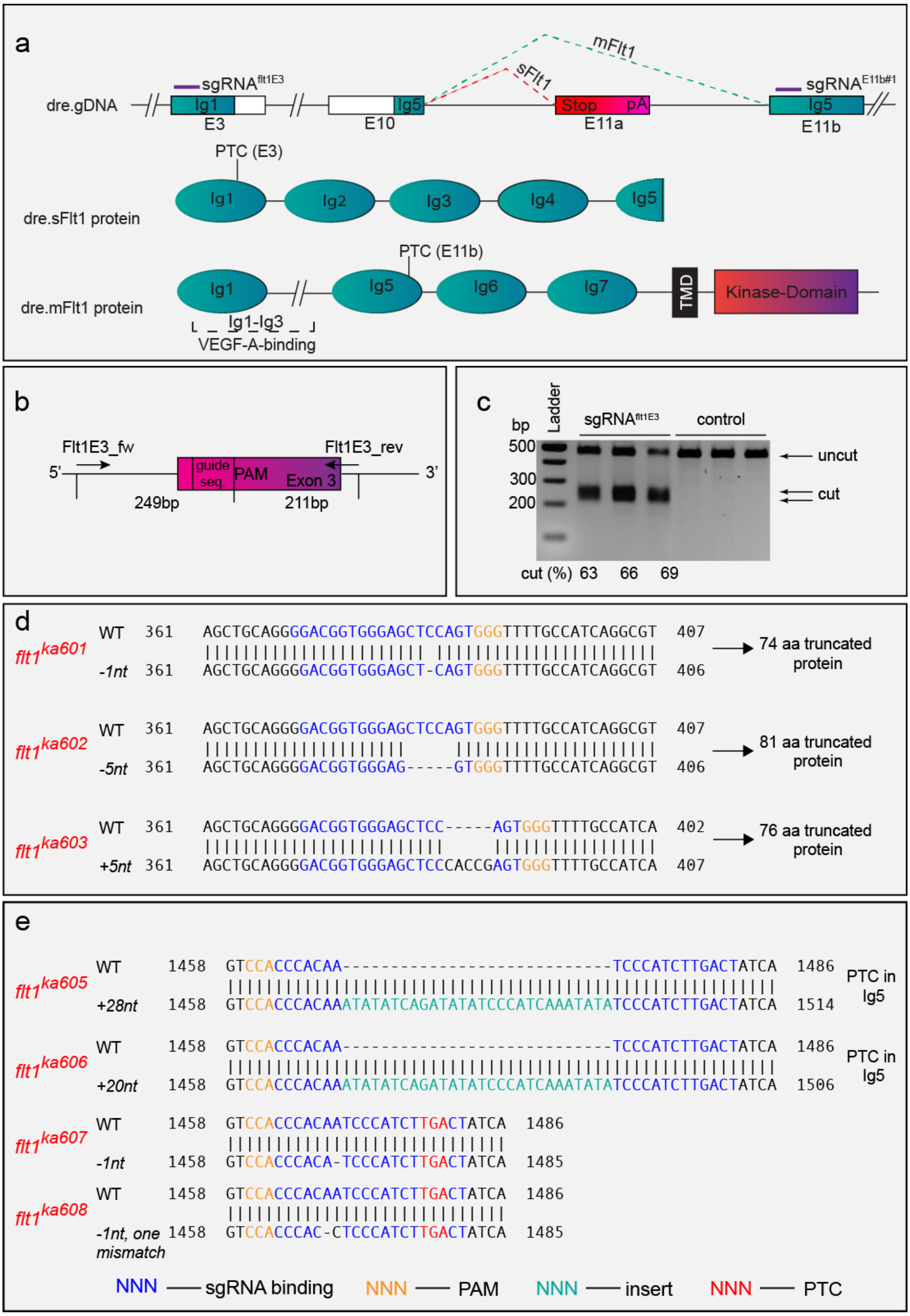

Supplementary Figure 2 | CRISPR/Cas9 approach for generating *flt1* mutants

(**a**) Flt1 exon structure and splice sites for zebrafish *sflt1* and *mflt1*. Schematic protein structure and IgG domains of sFlt1 and mFlt1 for comparison. Domains targeted by sgRNA in exon 3 (*full flt1* mutants *flt1*$^{ka601-603}$, targeting both *sflt1* and *mflt1*) and exon 11b (targeting only *mflt1, flt1* $^{ka605-608}$) are indicated. (**b**). Position of guide sequence, Flt1 exon 3 forward and

reverse primers and expected PCR band size after T7EI cleavage. (**c**) T7EI assay and quantification of $sgRNA^{flt1E3}$ (targeting exon 3) efficiency (**d**) Structure and DNA sequence of $flt1^{ka601}$ (-1nt), $flt1^{ka602}$ (-5nt), $flt1^{ka603}$ (+5nt) mutant alleles. (**e**) Structure and DNA sequence of *mflt1* mutants, $flt1^{ka605}$ (+28nt), $flt1^{ka606}$ (+20nt), $flt1^{ka607}$ (-1nt), and $flt1^{ka608}$ (-1nt, one mismatch) mutant alleles. PTC, premature termination codon; PAM, protospacer adjacent motif; sgRNA, small guide RNA.

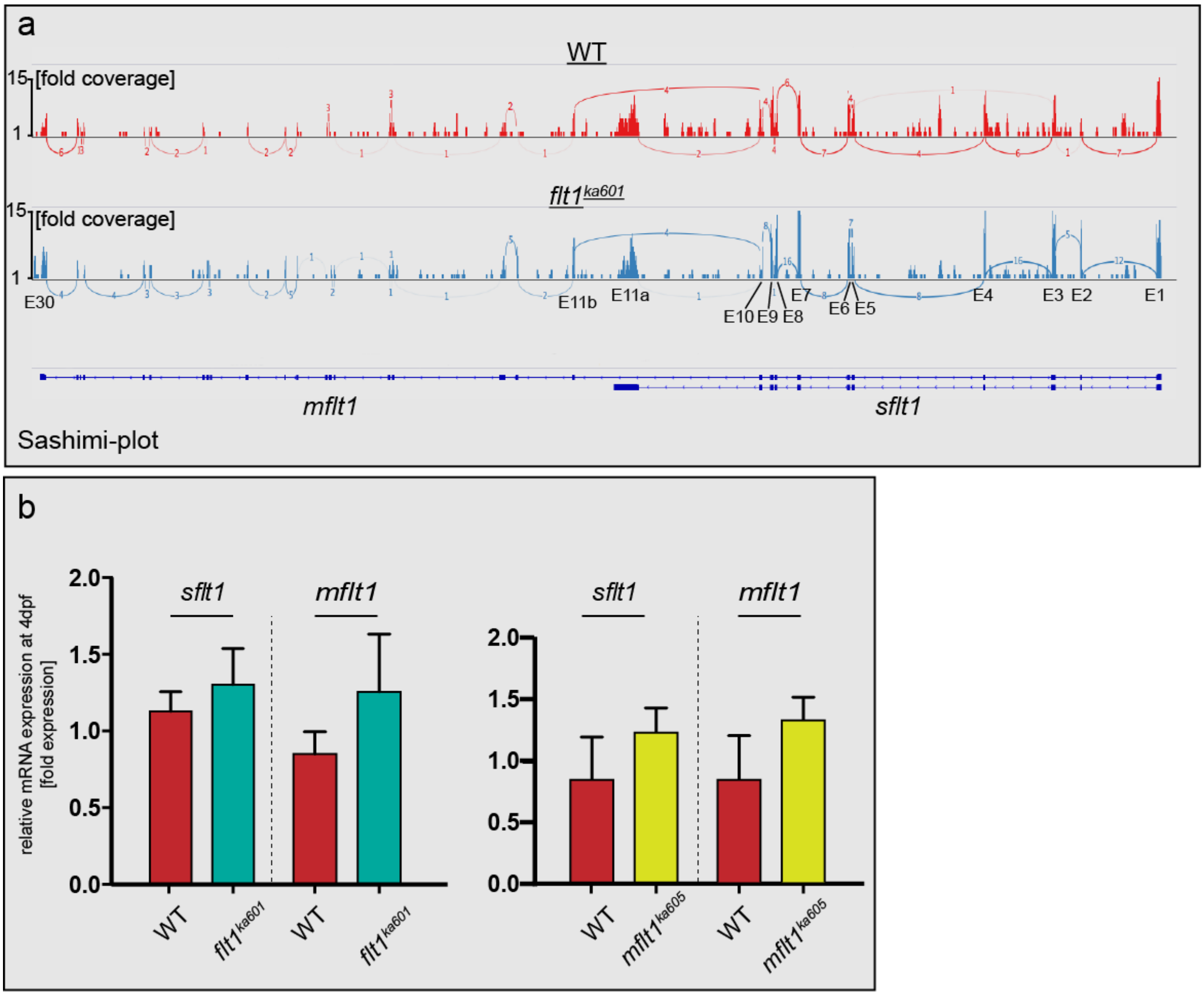

Supplementary Figure 3 | Analysis of non-sense mediated decay in *flt1* mutants

(**a**) Sashimi plot based on RNA-seq data of *flt1*^{ka601} mutant. The *sflt1* and *mflt1* intron-exon structures are indicated in the bottom panel of the graph; the number of corresponding reads for each exon (for WT in red, for *flt1*^{ka601} mutant in blue) presented in the upper part respectively. The read numbers are indicated (fold coverage). Exon-spanning reads are indicated by the arc symbol. Note that read numbers per exon in WT and *flt1*^{ka601} mutant are comparable. (**b**) PCR for *sflt1* and *mflt1* in the *flt1*^{ka601} (full mutant, left panel) and the *flt1*^{ka605} mutant (*mflt1* specific mutant, right panel). In both mutants we observed expression of *sflt1* and *mflt1*, at levels comparable to WT; mean ± s.e.m, n=3 experiments.

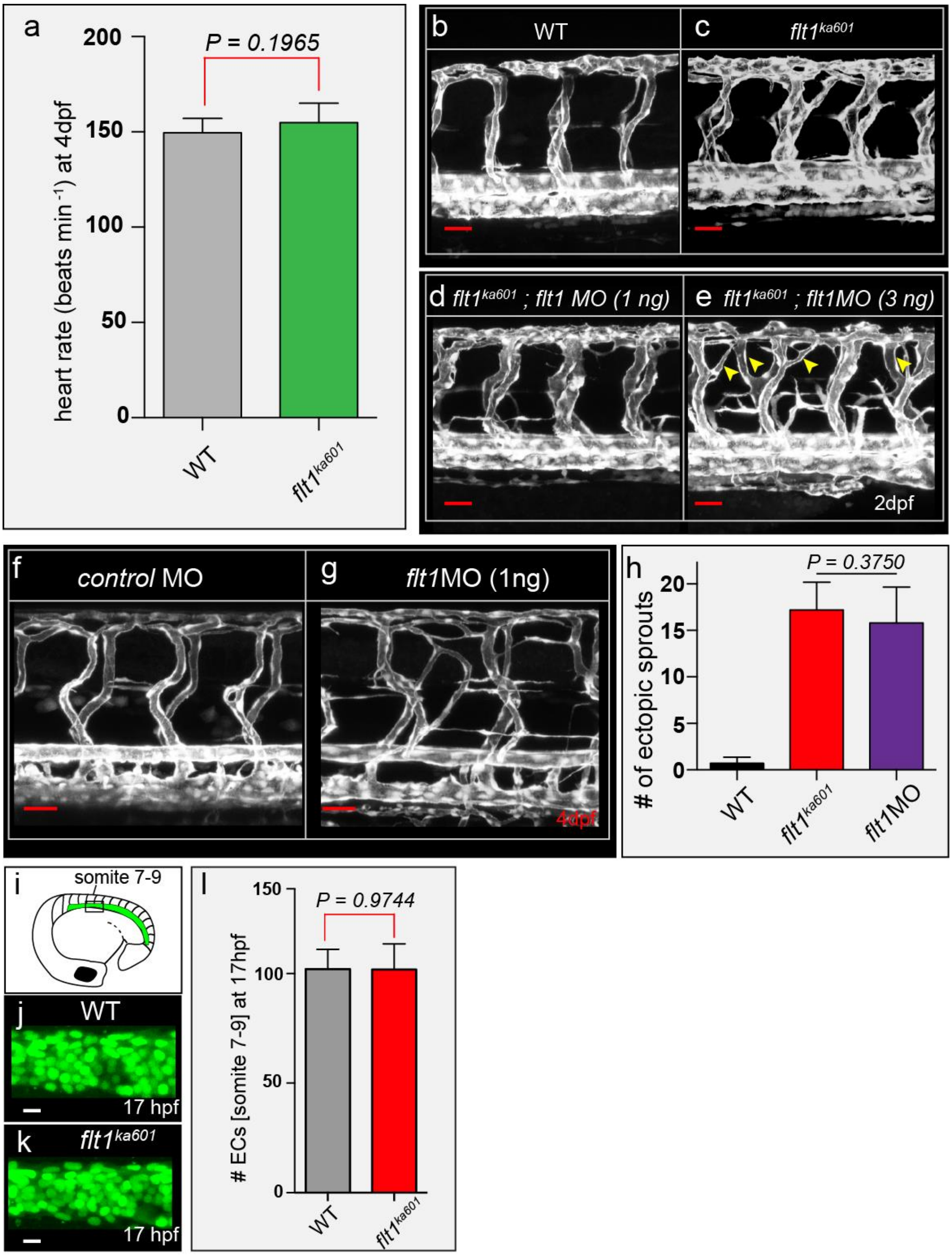

Supplementary Figure 4 | *Flt1* targeting morpholinos and quantification of endothelial cell numbers

(**a**) Heart rates in 4dpf WT and *flt1*^{ka601}, mean ± s.e.m, *n=10*. (**b-e**) *flt1*^{ka601} with 1ng (d) and 3ng (e) *flt1* targeting MO injected. Note: the 3ng dosage causes arterial branching defects (arrowheads) not observed *in flt1*^{ka601} mutants. (**f,g**) Vascular pattern in *Tg(kdrl:EGFP)* embryos injected with control MO (f), and 1ng *flt1* ATG targeting MO (g). (**h**) Quantification of

f,g; mean ± s.e.m, n=10. Note hyper-branching equal to *flt1*^{ka601}. (**i-k**) Imaging of endothelial nuclei in 17hpf WT and *flt1*^{ka601} embryos. EC numbers were counted between somite 7-9 (i). (**l**) Quantification of j,k shows no difference in EC numbers. ECs were counted using ImageJ plugin 3D object counter, mean ± s.e.m, *n=4*. MO, morpholino. Scale bar, 50µm in b-g; 10µm in j,k.

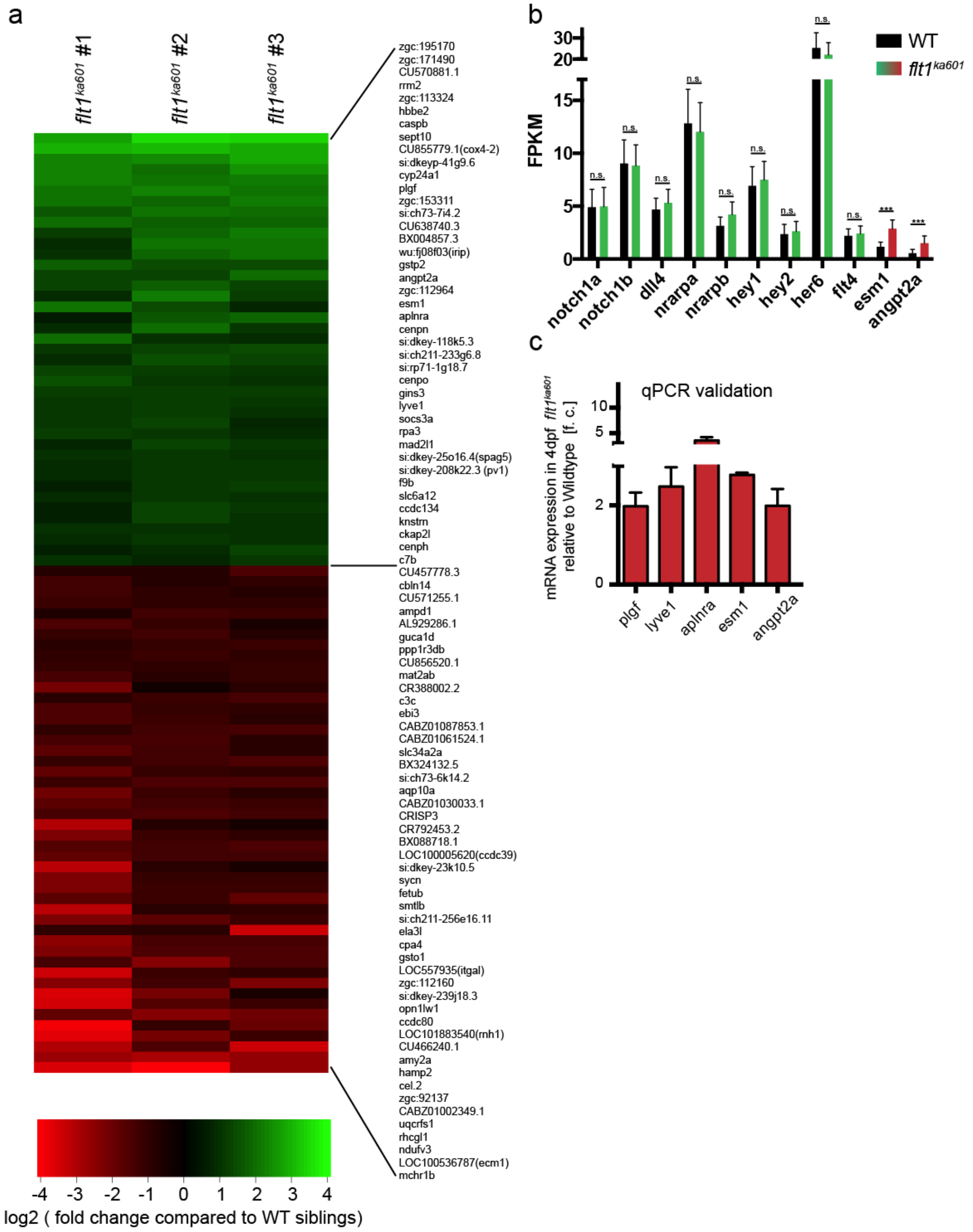

Supplementary Figure 5 | RNA sequencing analysis and qPCR validation of tip-stalk markers

(**a**) Heat map showing deregulated genes defined as ≥ 2 fold significantly regulated *(P< 0.05)* with two independent methods (DEseq and Cuffdiff) from three independent biological replicates. Upregulated genes in green, downregulated genes in red. (**b**) Expression of tip-

stalk cell markers *notch1, notch1b, dll4, nrarpa, nrarpb, hey1, hey2, her6, flt4, esm1* and *angpt2a* based on RNA-seq data in (a). Note: the Dll4-Notch signaling related genes (in green) were not deregulated but *esm1* and *angpt2a* (in red) were significantly upregulated. (**c**) qPCR validation of *esm1, angpt2a, aplnra, plgf*, and *lyve1*, identified as differentially expressed between WT and $flt1^{ka601}$ mutants in the RNA-seq analysis at 4dpf (see a, upper part heatmap, upregulated genes in green). Mean ± s.e.m, 3 experiments, Mann Whitney U-test.

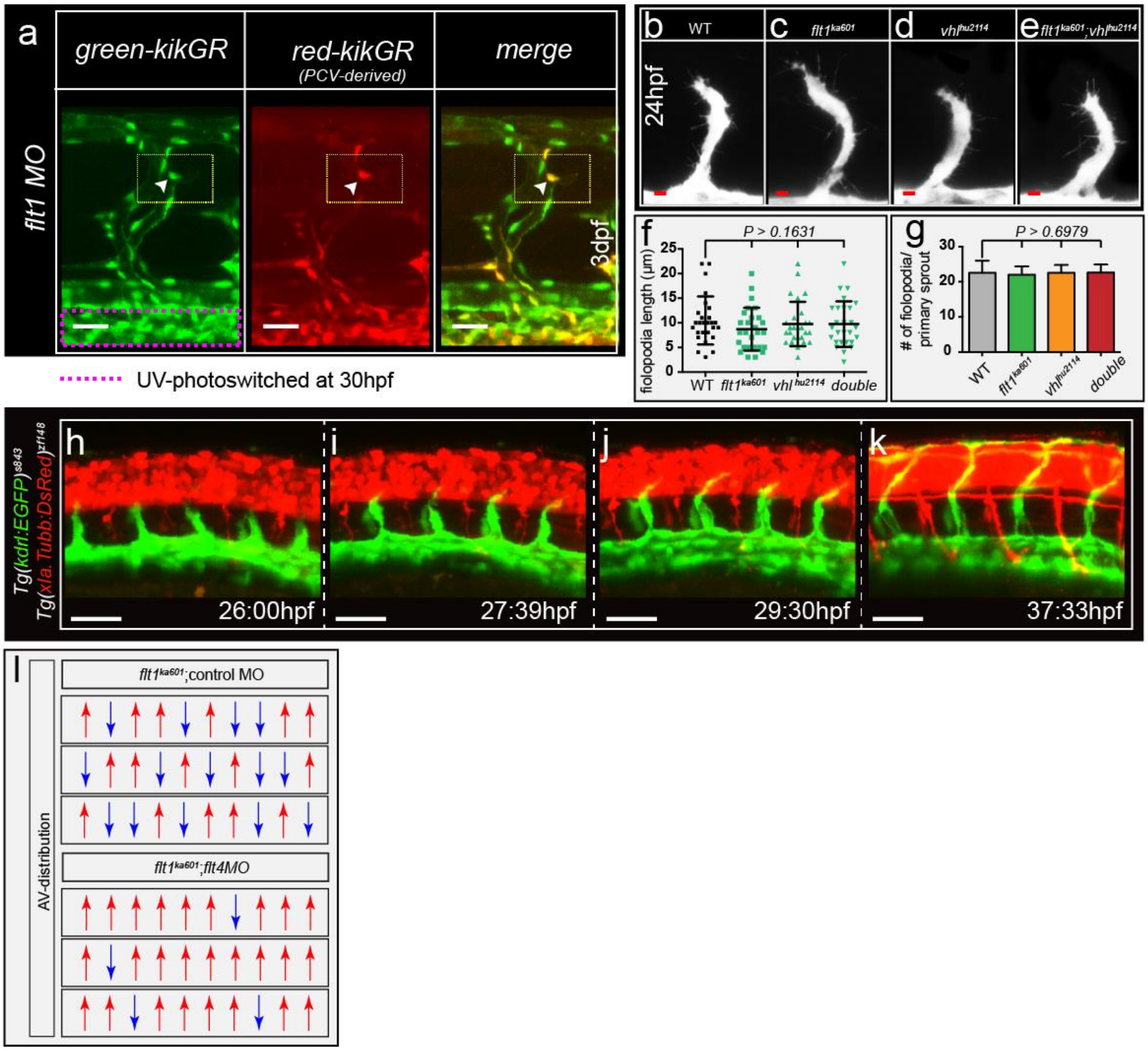

Supplementary Figure 6 | Endothelial cell behaviors in *flt1* loss-of-function and *vegfaa* gain-of-function scenarios

(**a**)*Tg(kdrl:nlskikGR)*^{hsc7} embryo injected with 1ng *flt1* morpholino. (left panel) Selected area in PCV (pink dotted box) was UV-photoswitched at 30hpf. (middle panel) Upon photo-conversion cells start to express the red reporter. PCV-derived cells migrate to the dorsal aspect of vISVs and contribute to venous ectopic sprouting upon loss of *flt1* at 3dpf (red cell in middle panel (arrowhead) contributes to ectopic sprouting - yellow-dotted box). (right panel) Merged image. (**b-e**) Confocal images of primary arterial segmental vessel sprouting in WT (b), *flt1*^{ka601} (c), *vhl*^{hu2114} (d), and *flt1*^{ka601};*vhl*^{hu2114} double mutants (e). (**f,g**) Quantification of filopodia characteristics for indicated genotypes. Note that there are no differences in sprouting or filopodia between indicated mutants and WT, mean ± s.e.m, *n=27* (f), mean ± s.e.m, *n=9* (g), t-test. (**h-k**) Time-lapse imaging of arterial sprouting in the developing

zebrafish trunk in *Tg(kdrl:EGFP)*^{s843}*, Tg(Xla.Tubb:DsRed)*^{zf148} double transgenic at indicated time points. Note sprouts develop in proximity to neural tube (j,k). (**l**) Characterization of intersegmental blood flow characteristics in *flt1*^{ka601} (l, top panel) and *flt1*^{ka601} injected with *flt4* targeting morpholino (l, bottom panel). In *flt1*^{ka601} mutant ISVs carry both arterial (red arrow up) and venous (blue arrow down) flow and the artery/vein ratio is about 1. Upon loss of *flt4*, almost all investigated ISVs carry arterial flow consistent with *flt4* blocking remodeling of arteries into veins. hpf, hours post fertilization. Scale bar, 25µm in a, h-k; 10µm in b-e.

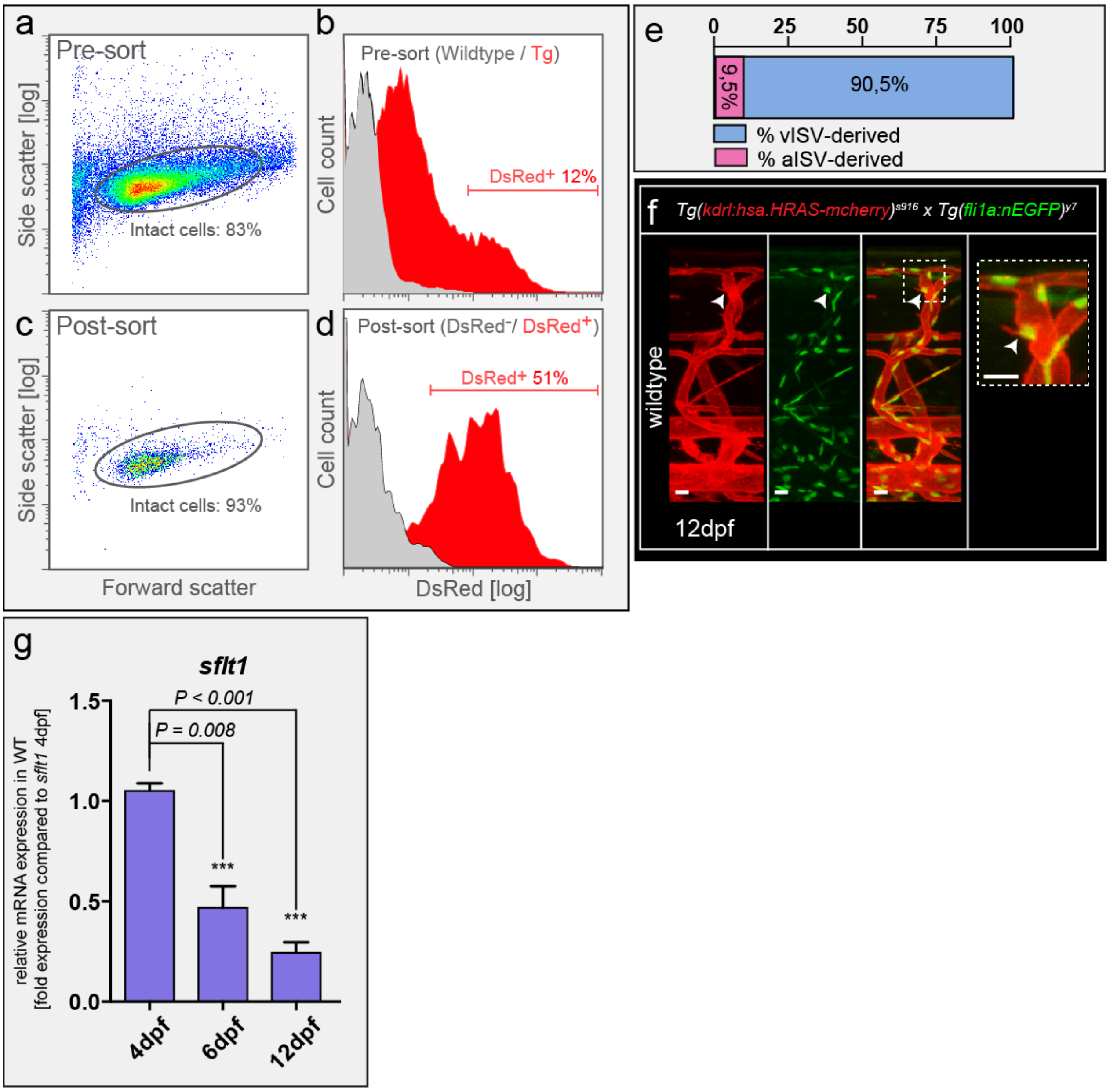

Supplementary Figure 7 | Spinal cord vascularization in WT zebrafish

(**a,b**) Neuronal cells were isolated from *Tg(Xla.Tubb:DsRed)*^{zf148} embryos at 3dpf by FACS with indicated gating settings. About 12% of all intact cells were DsRed+ neurons prior to sorting (Pre-sort). (**c,d**) Post-sorting analysis showed that sorted neuronal cells are enriched to 51% neuronal DsRed^{+} cells. DsRed^{-} cells contained less than 1.7 % DsRed^{+} cells. (**e**) Percentage of aISV and vISV giving rise to sprouts at level of neural tube in late stage WT embryo; 400 ISVs in n=20 embryos. (**f**) Nuclear positioning in sprout contributing to spinal cord vascularization in wildtype *Tg(kdrl:has.HRAS-mcherry)*^{s916};*Tg(fli1a:nEGFP)*^{y7} at 12dpf;

representative image from 6 embryos (**g**) Quantitative PCR for *sflt1* at indicated time points. Note decreased expression of *sflt1* associates with sprout appearance and spinal cord vascularization. mean ± s.e.m, n=3 experiments, 30 embryos/experiments dpf, days post fertilization; ISV, intersegmental vessel (a – artery, v – vein). Scale bar, 10µm in f.

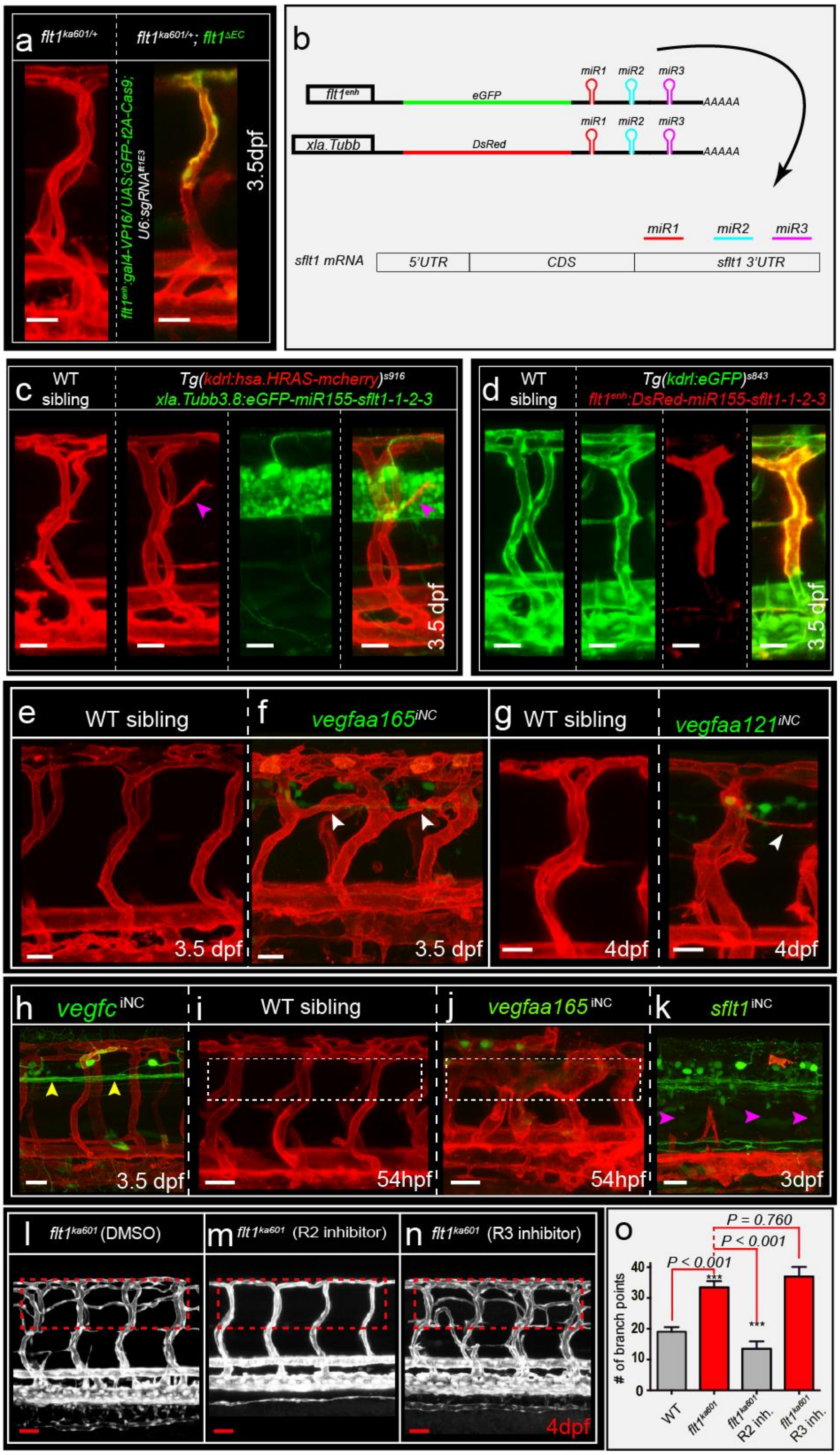

Supplementary Figure 8 | Targeting neuronal and vascular Flt1 and Vegfaa

(**a**) Vascular-specific depletion of *flt1* (*flt1*$^{\Delta EC}$) in *flt1*$^{ka601/+}$ heterozygous animals does not induce ectopic venous sprouting. Embryos are in *Tg(kdrl:has.HRAS-mcherry)*^{s916} background. (**b**) Graphical illustration of multiplex miRNA construct with GFP or DsRed

reporter, using miR-155 backbone coupled to three custom made miRNAs directed against the 3’UTR of *sflt1*. (**c**) Neuron-specific targeting of *sflt1* with miRNA approach induced ectopic venous sprouting in WT. 9 out of 12 vISVs with high miRNA expression in the adjacent neuronal cells (in green) formed sprouts. (**d**) Vascular-specific targeting of *sflt1* with miRNA approach failed to induce ectopic sprouting. vISVs expressing the miRNA construct (in red) did not induce sprout formation n=21. (**e,f**) Endoxifen inducible neuron specific *vegfaa165* gain-of-function induced at 52hpf after AV remodeling induces hyper-branching (arrowheads). (**g**) Endoxifen inducible neuron specific *vegfaa121* gain-of-function induced sprouting (arrowhead). (**h**) Endoxifen inducible neuron specific *vegfc* gain-of-function (yellow arrowheads) induced at 52hpf does not induce ectopic sprouting at level of neural tube. (**i,j**) Endoxifen inducible neuron specific *vegfaa165* gain-of-function induced at 30hpf, before completion of AV differentiation induces severe thickening of ISVs and abnormal vascular remodeling; compare dotted box in (i) and (j). (**k**) Constitutive (non-inducible) neuron-specific *sflt1* gain-of-function blocked ISV development; most ISVs were missing (arrowheads). (**l-n**) $Flt1^{ka601}$ mutant treated with DMSO (l); treated with Kdrl receptor signaling inhibitor ki8751 (m, R2 inhibitor); treated with Flt4 tyrosine kinase inhibitor MAZ51 (n, R3 inhibitor). (**o**) Quantification of l-n. Mean ± s.e.m, n=11/group, t-test. iNC, inducible, neuronal cell specific gain-of-function; R2, VEGF receptor 2; R3, VEGF receptor 3. miR, microRNA. Scale bar, 25μm in a, c-n.

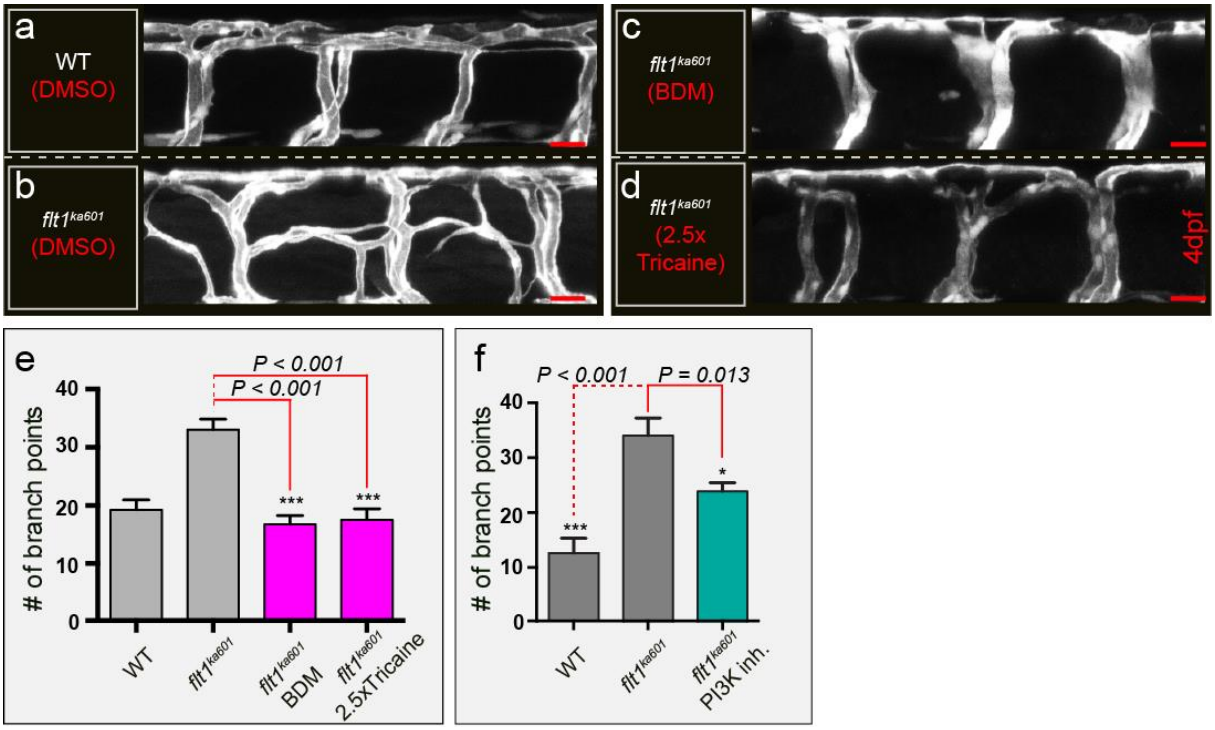

Supplementary Figure 9 | Ectopic venous sprouting requires hemodynamic factors and Akt

(**a-d**) Loss of hemodynamics rescues hyper-branching in *flt1*^{ka601} mutants. (a) WT treated with DMSO; (b) *flt1*^{ka601} treated with DMSO; (c) inhibition of cardiac activity with 2,3-Butanedione monoxime (BDM) in *flt1*^{ka601} (d) *flt1*^{ka601} exposed to 2.5X tricaine. mean ± s.e.m, 20-25 embryos/group, Mann Whitney U test. (**e**) Quantification of a-d. Note that inhibiting blood flow with BDM or tricaine inhibited ectopic branching. (**f**) Vascular branching after blockade of PI3 kinase with wortmannin. mean ± s.e.m, 3 experiments, 15-18 embryos/group, Mann Whitney U test. Scale bar, 20µm in a-d.

Supplementary Table 1 | Real-time qPCR and Taqman primer sequences

gene	name	forward primer	reverse primer
vegf aa	zvegfaa-E4-E5	5'-CAACGCGTATCGCAGCATAA-3'	5'-TGCCTTTGGCCTGCATTC-3'
vegf ab	zvegfab-E3-E4	5'-TGCTGGGTGCTGCAATGAT-3'	5'-CTCTTAATCTCCAAGGTAATGTTGTATGTG-3'
ef1a	zef1a-E4-E5	5'-GTTGCCTTCGTCCCAATTTC-3'	5'-CAATCTTCCATCCCTTGAACCA-3'
mflt1	zflt1-E19	5'-GTGAACACAAGGCTCTAATGACAGA-3'	5'-TGCGCCGAGGAGATTGAC-3'
sflt	z-sflt-E11a	5'-TCCGTCCCAATTTACCATTCC-3'	5'-TCTTGGGTGGCTGGATGAG-3'
plgf	z-plgf-E4-E6	5'-CACAAAGCCTGTGAATGTAGACT-3'	5'-TTCTCCTTCCTTTTTCTCCCTCTAT-3'
kdrl	zkdrl-E6-E7	5'-CAATGGCAGGATTCACTTTGAG-3'	5'-GACCGGTGTGGTGCTAAAATG-3'
kdr	zKdr-E12-E13	5'-ACAGGTGCATCGCTACCAATAA-3'	5'-GGACGCTTAGGTTGAGAAAACG-3'
lyve1a	zlyve1a_E4/5-E5/6	5'-GGCTCCACTGAAGCTGTTCC-3'	5'-GCCTTGCAGGGTCTTTTCGT-3'
angpt2a	zanpt2a-E4/5-E6	5'-TGTGACAAGGCAAGGTAGCAA-3'	5'-GTCCCCATGTCACAGTAGGC-3'
aplnra	zaplnra_E1	5'-GGACAAAACTCTGGGGGTGAA-3'	5'-ACACTCGCATCCACTCATCG -3'
esm1	zesm1_E2-E2/3	5'-TTGTGACAGAGAAACCGGCG -3'	5'-AACCCACTTCATTACCTGCTTCA-3'
hbbe2	zhbbe1_E1/2-E2	5'-ACTGCAGAGGGCTTTGATTGT-3'	5'-TGGCCTCAGCATTGTACAGG-3'

Supplementary Table 2 | Taqman probes

gene	name	taqman probe
vegfa a	zvegfaa-E4-E5	FAM-5'-TCAGCTGAGTTTCACAGAACACACCAAGTG -3'-TAMRA
vegfa b	zvegfab-E3-E4	FAM-5'- AAATGATGGAATGCACCCCCACC -3'-TAMRA
ef1a	zef1a-E4-E5	FAM-5'-ATGTTTGAGCTGGCCTCCAGCATGTT-3'-TAMRA
mflt1	zflt1-E19	FAM-5'-TGAAGATTCTCAATCATATAGGTCACCACATCAATG-3'-TAMRA
sflt	z-sflt-3'UTR	FAM-5'-TCTCTCACCTGTCAAATAACCCAACAACCG-3'-TAMRA
plgf	z-plgf-E4-E6	FAM-5'-ACCTACAACAAAACAAGACAGATGGAAACCCAGA-3'-TAMRA
kdrl	zkdrl-E6-E7	FAM-5'-AGTTTCATAAGGAGCGGATCAATCG-3'-TAMRA
kdr	zKdr-E12-E13	FAM-5'-GTTACTTGAAACACAATGACTCGCTG-3'-TAMRA

Supplementary Table 3 | Taqman ABI primer mixes

gene	ABI primer mix
vegfc	Dr03146062_g1
elav13 (HuC)	Dr03131532_m1
flt4	Dr03138041_g1
Unc55 (Unc5b)	Dr03430563_m1
Netrin1a	Dr03073979_m1
Semaphorin3aa	Dr03105514_m1
PlexinD1	Dr03203243_s1
Neuropilin1a	Dr03106127_m1

Supplementary Table 4 | sgRNA sequences

sgRNA sequence (without PAM)
sgRNAflt1E3: 5'-GGGACGGTGGGAGCTCCAGT-3'
sgRNAflt1E5: 5'-GGAATATCATCTGGAACAGC-3'
sgRNAflt1E11#1: 5'-GGCAGTCCAGGACGAAGGAGG-3'
sgRNAflt1E11#2: 5'-GGTGATGGTCAAGATGGGATTG-3'
sgRNAflt1E11#3: 5'-GGTCAAGATGGGATTGTGGG-3'
sgRNAflt1E11#4: 5'-GGAGAAGCCTCCTCCTTCGTCC-3'
sgRNAflt1E11#5: 5'-GGATGGTCAAGATGGGATTGT-3'

Supplementary Table 5 | sgRNA oligos for oligo-cloning into DR274

oligo1	oligo2
Flt1_E3_sgRNA_1+: 5'-TAGGGACGGTGGGAGCTCCAGT-3'	Flt1_E3_sgRNA_1-: 5'-AAACACTGGAGCTCCCACCGTC-3'
Flt1_E5_sgRNA_1+: 5'-TAGGAATATCATCTGGAACAGC-3'	Flt1_E5_sgRNA_1-: 5'-AAACGCTGTTCCAGATGATATT-3'
Flt1_E11b_sgRNA_1+:5'-TAGGCAGTCCAGGACGAAGGAGG-3'	Flt1_E11b_sgRNA_1-: 5'-AAACCCTCCTTCGTCCTGGACTG-3'
Flt1_E11b_sgRNA_2+:5'-TAGGTGATGGTCAAGATGGGATTG-3'	Flt1_E11b_sgRNA_2-: 5'-AAACCAATCCCATCTTGACCATCA-3'
Flt1_E11b_sgRNA_3+: 5'-TAGGTCAAGATGGGATTGTGGG-3'	Flt1_E11b_sgRNA_3-: 5'-AAACCCCACAATCCCATCTTGA-3'
Flt1_E11b_sgRNA_4+: 5'-TAGGAGAAGCCTCCTCCTTCGTCC-3'	Flt1_E11b_sgRNA_4-: 5'-AAACGGACGAAGGAGGAGGCTTCT-3'
Flt1_E11b_sgRNA_5+: 5'-TAGGATGGTCAAGATGGGATTGT-3'	Flt1_E11b_sgRNA_5-: 5'-AAACACAATCCCATCTTGACCAT-3'

Supplementary Table 6 | Primer sequences used for cloning and genotyping

primer name	primer sequence
pME_GFP_p2A_fw	5'-GCAGGAGACGTGGAGGAGAACCCTGGACCCGGGGAATTCAAGGCCTCTCGAGCCTCTAGAT-3'
pME_GFP_p2A_rev	5'-TTGCTTTAACAGAGAGAAGTTAGTAGCTCCGCTTCCTGAATTCCCAGATCTTCCACCGCC-3'
vegfc_p2A_fw	5'-ATCAGCGCTCACTTATTTGGATTTTCTGTC-3'
vegfc_p2A_rev	5'-AGTCTCGAGTTAGTCCAGTCTTCCCCAGTATGTG-3'
sflt1_p2A_fw	5'-ATGTTCGATATATTATTTGTGATGATATTTGG-3'
sflt1_p2A_rev	5'-AAGTCTCGAGTCAGGCCAGCCGCGCCGGG-3'
Vegfaa_p2A_fw	5'-AACTTGGTTGTTTATTTGATACAGTTATTTCTCGC-3'
Vegfaa_p2A_rev	5'-AGTCTCGAGTCATCTTGGCTTTTCACATCT-3'
U6_flt1E3_1	5'-GGGACGGTGGGAGCTCCAGTGT-3'
U6_flt1E3_2	5'-ACTGGAGCTCCCACCGTCCCGA-3'
Flt1_E3_gDNA_f	5'-CAGCTCAACACACACAGTATTGTTTTA-3'
Flt1_E3_gDNA_r	5'-ACACCTGAAGCATCTTACCTGTGA-3'
Flt1E11A2386576F	5'-ATTCCCAAGAGACCTGAAATCGGAA-3'
Flt1E11A2386151R	5'-GCTTGATTGCAGTTATCTTGAGGCA-3'

Supplementary Table 7 | miRNA155 sflt1-3'UTR specific target sites

target site	sequence (mmu miR-155 loop in red)
sflt1 3'UTR-1	5'-TGAAGACGGAGGGACAATCACGTTTTGGCCACTGACTGACGTGATTGTCTCCGTCTTCA-3'
sflt1 3'UTR-2	5'-AATAGATCAAGCTCCTGAGGAGTTTTGGCCACTGACTGACTCCTCAGGCTTGATCTATT-3'
sflt1 3'UTR-3	5'-TAGAGATTGAGGCTTGGTTCAGTTTTGGCCACTGACTGACTGAACCAACTCAATCTCTA-3'
